# One Health education in Kakuma refugee camp (Kenya): From a MOOC to projects on real world challenges

**DOI:** 10.1016/j.onehlt.2020.100158

**Published:** 2020-08-20

**Authors:** Isabelle Bolon, Jade Mason, Paul O'Keeffe, Philippe Haeberli, Hassan Abdi Adan, Joel Makamba Karenzi, Ali Abdirahman Osman, Samuel Mwangi Thumbi, Veronicah Chuchu, Mutono Nyamai, Sara Babo Martins, Nadja C. Wipf, Rafael Ruiz de Castañeda

**Affiliations:** a Institute of Global Health, Department of Community Health and Medicine, Faculty of Medicine, University of Geneva, Campus Biotech, Chemin des Mines 9, CH-1202 Geneva, Switzerland; b InZone, University of Geneva, Global Studies Institute, Sciences II, 30, Quai Ernest Ansermet, CH-1211 Geneva 4, Switzerland; c Pôle de soutien à l'enseignement et l'apprentissage, University of Geneva, Rue du Conseil-Général 10, CH-1205 Geneva, Switzerland; d Kakuma Refugee Camp, Kenya; e Institute of Tropical and Infectious Diseases, University of Nairobi, P.O. Box 19676, 00202 Nairobi, Kenya; f Paul G Allen School for Global Animal Health, Washington State University, Allen Center, P.O. Box 647090, 1155 College Ave., Pullman, WA 99164, USA; g Department of Public Health, Pharmacology and Toxicology, University of Nairobi, P.O. Box 29053, 00625 Nairobi, Kenya; h Washington State University – Global Health Program, Institute of Tropical and Infectious Diseases, P.O. Box 19676, 00202 Nairobi, Kenya; i Vector Control Group, Swiss Tropical and Public Health Institute, Socinstrasse 57, P.O. Box, 4002, Basel, Switzerland; j University of Basel, Petersplatz 1, P.O. Box, 4001, Basel, Switzerland

**Keywords:** One Health, Global Health, MOOC, Blended learning, Project-based learning, Refugee camp

## Abstract

Today, the world counts millions of refugees but only a fraction of them have access to higher education. Despite the multiple public health problems in refugee camps and the need to build local capacities to prevent and combat them, University level courses in public health are largely unavailable for refugees. This paper describes the development, implementation and evaluation of an innovative two-module blended-learning programme on One Health in Kakuma refugee camp (Kenya). This programme combines: (I) Interdisciplinary and multi-expert MOOC on “*Global Health at the Human-Animal-Ecosystem interface*”; (II) peer-to-peer learning involving students from University of Geneva Master of science in Global Health and research collaborations around specific and locally-relevant problems; (III) online mentoring and lecturing by experts from the Institute of Global Health of the University of Geneva in Kakuma. A total of 67 refugees applied to Module 1; 15 started the Module 1 in October 2017, of these 14 completed it and 6 passed the exams, finally five students started the Module 2 in October 2018 which they all passed in February 2019. Five student-led collaborative projects were developed focusing on the conception of a community-based monitoring system for prevalent diseases in the camp. With such a pedagogic approach, the programme provides an overview on Global Health challenges at the human-animal-ecosystem interface and the importance of the One Health approach, and introduces students to scientific research through interdisciplinary and international collaborations and innovation. The high number of applicants and positive feedback from students in Kakuma show the interest in One Health education in the camp. This learning experience ultimately aims at building local knowledge and capacity fostering “One Health” champions to reinforce local and national health system. This framework for One Health education could be potentially scaled up to other camps in Africa and the world.

## 1 Introduction

Today, 70.8 million people have been displaced globally because of persecution, conflicts, environmental and other disasters, and, among them, 25.9 million are refugees [1]. On average, refugees spend 20 years in exile [2] and only 3% have access to higher education [3]. The potential of this often young, skilled and motivated population remains largely neglected, yet mobile technologies and internet penetration have brought important opportunities [4,5].

MOOCs (Massive Open Online Courses) provide free and high-quality education to the millions of individuals across the world not able to attend a traditional campus course. Although MOOCs have mostly attracted people who live in developed countries [6], they also offer learning opportunities to underserved regions of the world [7]. MOOCs are currently used in refugee camps in Jordan, Kenya, Lebanon, and Turkey (e.g., Edraak, Jamiya Project, Kiron Open Higher Education, and InZone) [[8], [9], [10], [11]] covering domains such as business, engineering, computer science, and social science. Yet, limitations in internet connectivity, learning infrastructure and equipment (e.g., computers), and digital literacy, together with a permanent social insecurity for learners, challenge the scale-up, sustainability and impact of many of these MOOC-based educational programmes [8]. Few studies have quantified the impact of these types of higher education online programmes [9]. For instance, the *MOOCs4inclusion* study funded by the European Commission assessed how MOOCs can empower migrants and refugees for better inclusion, re-engagement in education and employment, and found that educational approaches that combine online and on-site teaching are more effective than ‘online only’ in fragile contexts [9]. Students with little digital literacy or formal education background need contact to real people in order to raise questions on difficult to understand concepts and for social networking [9]. In addition, students in refugee camps need context-specific learning support and academic guidance to gain relevant knowledge and skills that could improve their quality of life and career prospects [8,10,12,13].

Despite the multiple public health challenges in refugee camps and the need to raise awareness, educate and build local capacity to prevent and control them, university level on-line courses in public health are currently unavailable, to our knowledge. Between 2009 and 2017, 364 outbreaks of often deadly infectious diseases (e.g., cholera, influenza) affected 108 refugee camps worldwide [14]. Cholera outbreaks struck Kakuma refugee camp (Kenya) in 2005, 2009, 2015, and 2017 [[15], [16], [17], [18]]. Malaria remains a major health concern in camps in tropical Africa and South-East Asia [[19], [20], [21]]. Overcrowding, inadequate sanitation, and poor access to basic health services create optimal conditions for pathogens to spread across camps [14,22]. This raises important concerns about the impact the ongoing COVID-19 pandemic is going to have in such settings [23]. Moreover, many camps, especially those in tropical Asia and Africa, host diverse venomous animals such as spiders, scorpions and snakes, which put refugees at risk of potentially life-threatening stings or bites [[24], [25], [26], [27], [28]].

Most of these diseases result from close socio-ecological connections between humans, animals and their environment (e.g., [[29], [30], [31]]) and can be better understood, prevented and controlled by using scientific knowledge from ecology, veterinary and human medicine and by applying a One Health approach [32]. The One Health approach, based on an ecological approach to public health and the interdependence of human and animal health and their surrounding environment, promotes systemic thinking and transdisciplinary collaborations including multiple sectors (e.g., human and animal health) and local communities. Although there is increasing national and international political recognition of this approach in the fight against antimicrobial resistance and emerging and endemic zoonoses (e.g., Tripartite Collaboration between WHO-FAO-OIE) [33], many initiatives struggle to get implemented at the national and local levels [[34], [35], [36]]. This is partly due to a lack of awareness on the active role that local communities can play to alert about problems and tackle them in collaboration with research institutions and public health authorities [37,38].

Our objective was to develop, implement and evaluate the first blended educational programme on One Health for refugees in Kakuma refugee camp in Kenya. This programme builds on a MOOC on One Health developed by the University of Geneva (UNIGE) and uses a project-based learning model bringing together students from the camp with students at UNIGE and University of Nairobi (UoN).

## 2 Material and methods

### 2.1 Kakuma refugee camp and InZone

Kakuma refugee camp (Kakuma hereafter) is located 600 km from Nairobi in the Turkana County, an arid area of northwest Kenya. It is one of the world's largest refugee camps hosting 193,429 refugees and asylum-seekers from over 19 countries of origin (as of November 2019; [39]).

Considering the numerous public health problems at the human-animal-ecosystem interface and the importance to raise awareness about these problems and to ultimately build local capacity to prevent and control them in Kakuma, the Institute of Global Health of the Faculty of Medicine at the UNIGE together with InZone, decided to develop a One Health educational programme in 2017. InZone is a centre for higher education in refugee contexts at the UNIGE [40]. Since 2010, InZone has offered context-specific credit-bearing courses on human rights, children's rights, ethics, and basic medical education to refugees in Kakuma [12]. These courses are built on MOOCs and delivered with continuous support to participating students by (I) other students and experts in Geneva via WhatsApp, (II) on-site refugee facilitators, and (III) experts that teach face-to-face in the camp at the end of the MOOC. All InZone activities are conducted with the assistance of the Education and Protection sector of the United Nations High Commissioner for Refugees (UNHCR), which operates the camp in collaboration with RAS, the Refugee Affairs Secretariat of Kenya. InZone has built a solar-powered learning hub in the camp and supports learners with on-site facilitation and the requisite IT infrastructure.

### 2.2 One Health programme: content and implementation

The eight-week long MOOC “*Global Health at the Human-Animal-Ecosystem Interface*” (MOOC hereafter) is at the core of the One Health programme. This MOOC was launched in March 2017 on *Coursera* with the contributions of 44 experts from 20 institutions. It provides an overview of how One Health is interpreted and applied to different health issues (e.g., antimicrobial resistance, rabies) and contexts of the world (see detailed syllabus in Appendix A).

The One Health programme is organised into two sequential and complementary modules. The first, introductory module prepared students for the second, more advanced module. Module 1 also served as selection for highly motivated students prior to their admission to Module 2. Both modules are blended, combining distance learning and face-to-face interactions with experts in Kakuma. Table 1 presents the sequence and content of Modules 1 and 2.

**Table 1:** Sequence and content of Modules 1 and 2 of the One Health programme implemented in Kakuma.

	Module 1^a^ Introduction to *Global Health at the Human-Animal-Ecosystem Interface*	Module 2 *Global Health at the Human-Animal-Ecosystem Interface*
Learning objectives	To learn concepts and methods from epidemiology, veterinary sciences, animal biology and ecology, anthropology, citizen sciences etc., explore and learn about some of the major and current Global Health Challenges at the Human-Animal-Ecosystem Interface, discover key national and international players in One Health, get prepared to follow the full MOOC	To be able to critically discuss the interdependence of human-animal-ecosystem health from the local to the global level, to justify the added value of integrated approaches to health (e.g., One Health), apply transdisciplinary and system thinking, justifying the role of different disciplines, sectors, and institutions to tackle complex global health issues, apply scientific and evidence-based thinking, and generate innovative ideas using current and emerging methods and tools

Distance learning		
Duration	6 weeks	8 weeks
Dates	16 October - 24 November 2017	5 November - 28 December 2018
Content	An adapted version of the MOOC: 11 video-lectures^b^, readings (key definitions and concepts, short scientific articles, etc), online practice quiz	Full MOOC on *Coursera*: 53 video-lectures, readings (short scientific articles, reports, etc), online practice quiz
Learning materials	A USB stick with video-lectures, readings and a 60 page course guide with detailed instructions for each week Access to the InZone learning hub computers twice a week	MOOC content online on Coursera and offline via a USB stick provided in advance Access to the InZone learning hub computers twice a week
Mentoring	Continuous interaction with a group of international and interdisciplinary students from the UNIGE's Master in Global Health (peer to peer learning approach) via WhatsApp	
Activities	- Discussion and debate on the topic of the week with MGH students via WhatsApp - Field work: meeting local health professionals, identifying local health threats, etc.	- Work in interdisciplinary teams on projects aiming at designing a surveillance system for health risks at the human-animal-ecosystem interface in Kakuma refugee camp (Kenya) (project-based learning). Each team included students from Kakuma, MGH and University of Nairobi^c^ - Students collaborated through continuous online exchanges via WhatsApp - Teams competed for a potential opportunity to validate their project idea in Kakuma (travel grant for one MGH student) - Best project proposal selection by Institute of Global Health experts based on relevance, originality, and feasibility

Face-to-face learning in Kakuma		
Duration	5 days	5 days
Date	5–9 February 2018	18–22 February 2019
Content	- On-site lecturing involving experts (IB, RRdC) - Interactive activities: discussions and group work on specific local health problems to further prepare students for a final exam. - Exam: an oral assessment of the knowledge on the content of the video-lectures and the presentation and assessment of the group work.	- On-site lecturing involving experts (IB, RRdC) -Interactive activities: discussions on theory and practice with a focus on projects - Field work related to the selected project led by a MGH student - Exam: final MOOC quiz on Coursera, oral exam assessing knowledge and critical thinking, project presentation and assessment
Total Duration	7 weeks	9 weeks
UNIGE credits	3 ECTS	6 ECTS

Abbreviations: MGH: UNIGE Master in Global Health, MOOC: MOOC “Global Health at the Human-Animal-Ecosystem interface”.

a Module 1 was advertised in the camp in September 2017 by InZone management team via word of mouth and WhatsApp and students were selected based on a motivation letter and a short essay about animal species present in the camp and the advantages and risks they pose for humans.

b Video-lectures selected from the full MOOC and discussing the role of One Health around emerging infections (e.g., Avian influenza, MERS), neglected tropical diseases (e.g., snakebite, zoonotic tuberculosis), biodiversity conservation, etc.

c Students enrolled in a Master in veterinary epidemiology or public health, in a PhD or a research project.

Students in Kakuma had online and on-site support throughout the programme. When following the adapted version of the MOOC (Module 1) or the full MOOC (Module 2), they interacted continuously via WhatsApp with a group of international and interdisciplinary students from the UNIGE's Master of science in Global Health (MGH hereafter) (peer-to-peer learning approach). MGH students were based in Geneva and followed in parallel the full MOOC on *Coursera*. Online exchanges between students in Kakuma and Geneva were guided and supervised by two experts at the UNIGE's Institute of Global Health (IB, RRdC). These experts supported students in Geneva through regular class meetings. They mentored students in Kakuma during the week of on-site lecturing in the camp. In Kakuma, three on-site facilitators supported students and ensured the smooth development of programme activities, while reporting progress to the team in Geneva (e.g., completion of quiz and field work).

### 2.3 Programme evaluation

At the end of Module 1, experts from the Institute of Global Health used an online anonymous survey to solicit feedback of students in Kakuma on their learning experience and future career perspectives. On-site course facilitators were also surveyed to assess their view on student motivation and practical challenges during the course. Survey questions are included in Appendix B.

At the end of Module 2, the MGH student who visited Kakuma (JM) conducted a focus group discussion in Kakuma with the five students who passed the 2-Module programme to assess, 1) their experience while following this programme; 2) their experience while interacting with the students in Geneva; 3) their experience with project-based learning, and 4) how they will move forward as One Health students.

## 3 Results

### 3.1 Students' engagement and completion of the programme

A total of 67 students in Kakuma applied to the One Health programme. Their motivations for enrolment are reported in Appendix C. To ensure close interaction with the students in Kakuma, a group of 15 was selected to start the Module 1 in October 2017. One student dropped out of the programme for unknown reasons. Six students out of the 14 who completed the Module 1 successfully passed it in February 2018 and accessed Module 2 in October 2018. Two students left the camp and one on-site facilitator joined this Module. The final number of students was five, who passed Module 2 in February 2019. The profile (i.e. gender, age) of applicants and of the students who were selected and passed the Module 1 are shown in Appendix C.

### 3.2 Programme activity

**Fig. 1:**
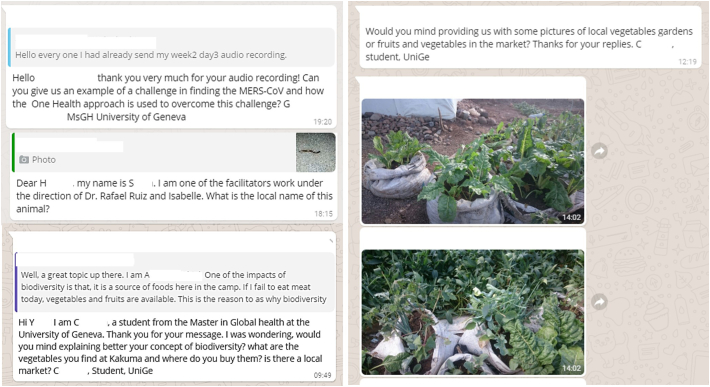
WhatsApp exchanges between students in Kakuma and MGH students.

A total of 26 MGH students (cohort 2017) were involved in Module 1 and 32 MGH students (cohort 2018) in Module 2. MGH students played an important mentoring role. They animated WhatsApp discussions by posting weekly questions and raising topics for debate, and by providing concept clarifications and responses to spontaneous questions posed by the students in Kakuma (Fig. 1). These questions often referred to general concepts and definitions (e.g., the difference between an emerging and an endemic zoonosis; the difference between an anthroponosis and a zoonosis), but also raised more challenging issues that required MGH students to do additional research to provide their more detailed responses (e.g., whether dog culling would be an effective strategy to tackle rabies in Kakuma, or how citizen science could be applied to tackling public health problems in Kakuma).

### 3.3 Projects

A total of five student-led projects were developed during Module 2 focusing on the conception of a community-based monitoring system for prevalent diseases in the camp: malaria, cholera, zoonotic food-borne diseases, rabies and snakebite envenoming (Table 2). Each group included one representative from Kakuma, 5–6 MGH students and one student from the UoN. Students in Kakuma were the project focal points in the field providing key information on the camp and the project feasibility, while students from the UoN provided specific technical expertise on the disease based on their own research experience in Kenya. MGH students coordinated the project and supported the research, tapping into the scientific literature and the network of international experts and organisations based in Geneva (e.g., WHO, MSF, UNHCR).

**Table 2:** Characteristics of the five research projects developed collaboratively by students from Kakuma, UNIGE Master in Global Health and University of Nairobi. The objective was to conceive a monitoring system for a vector borne disease, a water borne disease, a zoonotic food borne disease, rabies and snakebite or scorpion sting in Kakuma refugee camp using context-specific and locally adapted tools (e.g., frugal innovation) and participatory methods (e.g., citizen science). These broad thematic areas were proposed to the students by experts at the Institute of Global Health of the UNIGE.

Title of the project	Objectives	Data to be collected	Community involvement	Tools to be used
Cholera Surveillance System in Kakuma Refugee Camp	A surveillance system that determines if the presence of *Vibrio cholerae* in the Tarach river and its tributaries contributes to the contraction of cholera	Detection of *V. cholerae* in the Tarach River and its tributaries; GPS data; behavioural data (cholera patients' interactions with the river)	Surveillance teams will include InZone students who will become mentors for community members	A rapid portable biosensor for field detection of *V. cholerae* in environmental water (OmniVis technology)
Integrated Surveillance System for Rabies (ISSR)	Establish household level incident-based reporting of dog bites by secondary school children as an early warning system for rabies exposure	Annual dog census; dog keeping knowledge, attitudes, and practices; dog bite incidence	Secondary school children and their teachers to report dog bite incidents and information about dog ecology and dog-keeping practices	Questionnaire administered through KoBo Toolbox
Community Based Surveillance System for Malaria in Kakuma Refugee Camp	Monitor malaria risk involving the community in identifying *Anopheles* larvae and mapping *Anopheles* breeding sites	Mapping of run-off pits; collecting, counting and identifying mosquito larvae in run-off pits	InZone students who will be trained to identify and collect mosquito larvae and who can be trained to become trainers of community members	UNHCR iMonitor application
Diarrhoea Incidence and Animal Proximity in Children Under Five Years in Kakuma Refugee Camp	To collect data for an early detection system of new diarrheal cases in children under 5 years old in Kakuma related to animal proximity and to animal-source food consumption	New cases of diarrhoea among children under 5; type of stool; contacts with animals/animal-source food; household characteristics (e.g., animal-keeping); pathogen detection in stool sample	10% of mothers with children under 5 years recruited as “surveillance officers” (SO) and trained by InZone students	Questionnaires will be completed weekly by SO via a mobile application
3S: Snakes, Surveillance and Smart tech	A community-based, risk assessment project for snakebites, measuring the distribution of snakes using a multi-layered real-time data collection method	Number of snake sightings; number of snakebites; environmental indicators (e.g., temperature, precipitation or floods); time, location of observations	“Snake Champions” found through InZone to motivate/promote people to use the App/SMS/calls.	Three-way data collection system: mobile phone App, SMS to collect descriptive data on the snake or call

Project proposals were evaluated by experts at the Institute of Global Health in December 2018 and the project on malaria (Table 2) was selected by consensus based on its relevance and feasibility. An MGH student (JM) received a grant from InZone to follow up on this project in Kakuma in February 2019. As we could support only one project, the other student project ideas were not further supported in the field in Kakuma.

### 3.4 Field validation of the project “Community Based Surveillance System for Malaria in Kakuma Refugee Camp”

**Fig. 2:**
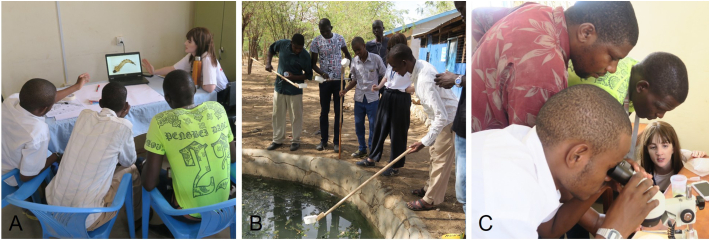
Introduction to mosquito larval morphology and key features for genus level identification (A). Field and lab work on mosquito larvae identification supervised by a MGH student in Kakuma (B-C) (Credit: I Bolon/InZone).

First, the MGH student received basic training on mosquito larvae collection and morphological identification at the Vector Control Group at the Swiss Tropical and Public Health Institute in Basel. Once in Kakuma, she trained students in these techniques during larval collection in the field and analysis in the lab using two stereomicroscopes that were brought to the camp (Fig. 2). The project was selected to be presented at the Global Compact on Refugees Academic Network Workshop convened by UNHCR in Geneva on November 13, 2019 and will be further developed by the MGH student during her 5-month internship at the Institute of Global Health.

### 3.5 Programme evaluation

At the end of Module 1, students in Kakuma and on-site facilitators were invited to complete an online anonymous satisfaction survey. A total of eight out of the 14 students in Kakuma who completed Module 1 and all three on-site facilitators completed the survey. When asked about their motivations to take Module 1: 4/8 students wanted to learn more about One Health and Global Health; 2/8 students were motivated by previous experiences in the field of infectious diseases (e.g., “*My motivation was instigated by the Anthrax outbreak in our location a decade ago*”, “*When I wanted to apply for this course I saw Ebola as one of the main topics at the Human-Animal-Ecosystem so I wanted to learn more about it*”); 2/8 students saw Module 1 as an opportunity to engage into community work (e.g., “*The need to feel the gaps in our community. These gaps include fighting malaria and snake bites among others*”). Concerning the course topics that were most interesting for the students: 5/8 students pointed to health problems associated with animals in the camp and in the world, as well as how these problems can be prevented (e.g., “*Zoonotic Tuberculosis due to consumption of unpasteurised dairy products won my heart …. I will use this knowledge to inform locals & families around Kakuma and beyond to stay aloft of the Zoonotic Tuberculosis by taking properly prepared dairy products, especially in the Camp and its environs*“). When asked about their future aspirations: 7/8 students were committed to championing the One Health approach in their communities (e.g., “*I would like to be a mobilizer in the One Health arena thus mobilizing all relevant disciplines in tackling a problem or to prevent the problem from even occurring”*). All the students would recommend the course to other refugees in Kakuma. On-site facilitators highlighted the motivation of the students (e.g., “*The level of student's motivation was high and excellent. [...] So it was quite encouraging*”) and their potential to apply the knowledge gained to actions in their community (e.g., “*Some students are active in the community and putting the concepts they learned into practice*”, “*The knowledge that they acquired from the MOOC is being shared in the community through health talk*”). Students also faced some practical challenges during the course including limited computer skills and internet access, and lack of smartphones in some cases, or limited access to InZone learning hub due to heavy rain and flooding.

During the focus group discussions, the five students that completed Module 2 reported that they enjoyed the MOOC content and the interaction with students in Geneva. Yet, two students felt that the group work was one sided, with students in Geneva leading the work and limiting the opportunities for contribution for the students in Kakuma. These students wanted to have more responsibility in the project, including, for example, writing parts of the project proposal. All students agreed on the importance of this course and believe that, as students, they could contribute to basic ecological and epidemiological field work activities, such as those associated with the malaria project. They are also keen to support this training programme by supporting future students. They suggested climate change and scorpion stings as further topics to be included in the programme. Detailed focus group feedback is included in Appendix D.

## 4 Discussion

To our knowledge, this is the first educational programme addressing One Health in a refugee camp combining a MOOC and context-specific project-based learning. Using a peer-to-peer learning model, this programme makes refugees part of an international and interdisciplinary community of students from UNIGE and UoN.

MOOCs are often new for refugees (i.e. most of the distance learning initiatives for refugees have been launched after 2014 [41]) and mentoring is crucial for their effective learning experience [8,9,12,42]. Students in Kakuma were mentored by MGH students through continuous online exchanges. The absence of an academic hierarchy and the fact that most students belonged to the same age group facilitated the ease of discussions, as also observed in other online peer-to-peer learning experiences [11]. Besides learning together about the content of the MOOC, interactions between these two groups of students were culturally enriching and illustrated the complexity of public health problems of the camp to those students based in Geneva.

A total of 67 refugees applied to this specialised programme, which is 2 to 3 times the numbers we observed for other InZone courses. These numbers could keep growing with a wider and multi-channel outreach strategy in the camp. Yet the number of applicants will always be relatively small since accessing higher education programs offered by InZone comes with certain requirements (having a secondary school diploma, proficiency in English, basic computer skills) that are only met by a minority of refugees.

This relative high number of applicants to our programme in Kakuma and the feedback from its participants highlight the interest in One Health education in the camp. With our programme students in Kakuma learned to look at public health problems in the camp and more widely at global health problems through a One Health lens (i.e. systemic and transdisciplinary thinking). Students were introduced to scientific reasoning and discovered new disciplines (e.g., entomology, epidemiology, anthropology), key national and international players in One Health (e.g., Tripartite collaboration: FAO, OIE, WHO) and reliable sources of information (e.g., WHO, Institute of Health Metrics and Evaluation). As part of their projects, they collaborated with other students of different origin and educational background from UNIGE and UoN, pushing all students to think about how to best share knowledge and combine their expertise. This exercise is central to One Health and can improve the originality of ideas and research approaches [43]. At the same time, this empowers students and gives them a sense of belonging to an academic community [37], especially because they developed relationships and discovered common interests with other students in Kakuma, Nairobi and Geneva. Altogether, our programme encouraged students in Kakuma to champion One Health and to help their community tackle health problems at the human-animal-ecosystem interface. Participatory approaches such as citizen science and mobile technologies discussed in this programme generated great interest among the students, who saw an opportunity to take relatively simple and inexpensive community-led initiatives that could generate local, national and internationally relevant data (e.g., reporting and photographing encounters with wild animals in the camp using mobile phones). Most course projects encouraged this type of participatory approach (Table 2) and with the project on “Community Based Surveillance System for Malaria in Kakuma Refugee Camp”, we showed that with some basic training, students can perform relatively simple but highly relevant tasks (e.g., collect larval samples from stagnant water ponds, search for mosquito larvae using a stereomicroscope and send photos of them to an entomologist) that could contribute to improve our understanding of malaria risk in the camp. Our students may well become a “task force” at the community level supporting local health and environmental stakeholders such as the International Rescue Committee who leads the health sector in Kakuma or the Norwegian Refugee Council who implements UNHCR's sanitation and hygiene programmes in the camp. Students could help in the collection of field data and performing tasks that are not generally done by their staff (i.e. due to time or budget constrains) and that can complement and benefit from existing initiatives in the camp. For example, *iMonitor*, an app developed by UNHCR for refugees to report infrastructural problems in the camp [44], could also be used by our students and more widely by the community in Kakuma to spot and alert about environmental hazards or risks associated with animals (e.g., stagnant water and mosquito breeding sites, snakes and snakebite). Yet implementing the growing number of relevant project ideas that emerge from our programme every year in Kakuma raises multiple challenges that go beyond their primary role as educational experiences for refugees. This includes questions around project leadership, ownership, funding, and sustainability, that still need to be carefully analysed, especially because of the complexity of the humanitarian context and the many ethical considerations around what refugees can and cannot do [45].

Our One Health programme also opens potential educational opportunities for students in Kakuma outside the camp, especially because this programme is part of UNIGE's continuing education and therefore students obtain 3 ECTS credits for Module 1 and 6 ECTS for Module 2. ECTS credits are recognised in all European Universities and in a growing number of African ones [46]. Yet continuing education and career development remain extremely challenging and uncertain for most refugees, who struggle to obtain requisite funding, equivalence for their foreign credentials, or visas to study in countries outside of their hosting country.

This programme was enriching for MGH students too, particularly because they were confronted with real world problems and with the multiple difficulties related to researching in a refugee camp [47]. Refugee camps are often “black boxes” and access to public health scientific literature is limited, which challenged our students and pushed them to think innovatively (e.g., using social media data and grey literature as sources of information). This also encouraged MGH students to collaborate with students in the camp and those at the UoN in an effort to gain a better understanding of the camp and related public health problems. They also developed their own network of experts from academic and humanitarian institutions in Geneva, which has the potential of generating future professional opportunities.

The programme proposed here provides a framework for One Health education in a refugee camp. This framework could benefit from other training programmes in One Health (e.g., [48]) or include other important topics like climate change [49] to keep evolving. It could be potentially scaled up to other refugee camps in Africa and the world.

## Declaration of Competing Interest

The authors have declared that no competing interests exist.

## Appendix Supplementary data

Appendix A Syllabus of the MOOC “*Global Health at the Human-Animal-Ecosystem Interface*” Image 1

Appendix B Survey of Kakuma Students and On-site Facilitators Image 2

Appendix C Motivations and Profiles of Applicants and Students Image 3

Appendix D Focus Group Discussion Image 4
