# Supplementary material for: One Health education in Kakuma refugee camp (Kenya): From a MOOC to projects on real world challenges
Source: One Health. 2020 Aug 20;10:100158. doi: 10.1016/j.onehlt.2020.100158 (PMC7439830; doi:10.1016/j.onehlt.2020.100158)
Supplement: Appendix A — Syllabus of the MOOC “Global Health at the Human-Animal-Ecosystem Interface” [file mmc1.pdf]

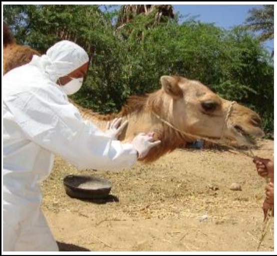

Global Health at the Human-Animal-Ecosystem Interface

General Introduction

Section 1 - Global Health at the Human-Animal-Ecosystem Interface: The Need for Intersectoral Approaches

Video-lecture	Expert and Affiliation
The importance of One Health for Global Health	Prof. Arnaud Fontanet<br>Director Center for Global Health<br>Head of Emerging Diseases Epidemiology Unit<br>Institut Pasteur, France
The Tripartite Collaboration: FAO-OIE-WHO	Dr. Kazuaki Miyagishima<br>Director, Department of Food Safety and Zoonoses<br>World Health Organization (WHO)
Cross-Sectoral, Multidisciplinary Risk Assessment for Health Threats at the Human-Animal Interface	Dr. Elizabeth Mumford<br>Human-Animal Interface<br>Department of Global Capacities, Alert, and Response<br>World Health Organization (WHO)
How to link Global Health Priorities with Biodiversity?	Dr. Maria Neira<br>Director<br>Public Health and the Environment Department<br>World Health Organization (WHO)
Global Animal Trade: Health Risks and Regulations	Dr. John Berezowski<br>Veterinary Public Health Institute<br>Vetsuisse Faculty<br>University of Bern, Switzerland
Sendai Framework for Disaster Risk Reduction & Health	Dr. Chadia Wannous<br>The United Nations Office for Disaster Risk Reduction (UNISDR)

Section 2 - Emerging Infectious Diseases

Video-lecture	Expert and Affiliation
Challenges for the Spread of Emerging Viruses	Prof. Laurent Kaiser<br>Head of the Division of Infectious Diseases<br>Director of the Laboratory of Virology<br>University Hospitals of Geneva (HUG)<br>University of Geneva, Switzerland
Investigating Emerging Zoonotic Outbreaks: Importance of the One Health Approach	Dr. Maria van Kerkhove<br>Head of the Outbreak Investigation Taskforce<br>Center for Global Health,<br>Institut Pasteur, France
Institute Pasteur Global Network & the fight against Emerging Infections	Dr. Marc Jouan<br>Director of the International Affairs Department<br>Institut Pasteur, France
IHR 3.0 Simulator: Improving our Response to Global Health Emergencies	Prof. Antoine Flahault<br>Director, Institute of Global Health<br>Faculty of Medicine, University of Geneva
Disease Ecology of Nipah Virus in Cambodia: Exploring Bat Population Dynamics at the Frontline of the Emergence	Dr. Julien Cappelle<br>Centre de coopération internationale en recherche agronomique pour le développement (CIRAD)<br>Institut Pasteur, Cambodia

Disease Ecology of Nipah Virus in Cambodia: Exploring the Human-Bat Interface	Dr. Julien Cappelle<br>Centre de coopération internationale en recherche agronomique pour le développement (CIRAD)<br>Institut Pasteur, Cambodia
Fight against VBDs: Global Change & the Need for an Interdisciplinary Approach	Prof. Antoine Flahault<br>Director, Institute of Global Health<br>Faculty of Medicine, University of Geneva, Switzerland
The Reversed Spill-over: From Humans and Domestic Animals to Wildlife	Dr. Geoffroy Mauvais<br>Program on African Protected Areas and Conservation (PAPACO)<br>International Union for Conservation of Nature (IUCN)
The Importance of Understanding Animal Behaviour for Global Health	Dr. Rafael Ruiz de Castañeda<br>Institute of Global Health<br>Faculty of Medicine, University of Geneva, Switzerland
Bushmeat & Emerging Infectious Diseases: Joint conservation & Health Strategies	Dr. Geoffroy Mauvais<br>Program on African Protected Areas and Conservation (PAPACO)<br>International Union for Conservation of Nature (IUCN)
Social Anthropology of Zoonoses	Dr. Tamara Giles-Vernick<br>Medical Anthropology and Environment Group<br>Institut Pasteur, France

**Section 3 - Antimicrobial Resistance & Zoonotic Foodborne Infectious Diseases**	
**Video-lecture**	**Expert and Affiliation**
The Global Burden of Antimicrobial Resistance	Dr. Elisabeth Tayler<br>AntiMicrobial Resistance Secretariat<br>World Health Organization (WHO)
WHO Global Action Plan on Antimicrobial Resistance	Dr. Elisabeth Tayler<br>AntiMicrobial Resistance Secretariat<br>World Health Organization (WHO)
Canadian Integrated Program for Antimicrobial Resistance Surveillance (CIPARS)	Prof. Andre Ravel<br>Faculty of Veterinary Medicine<br>University of Montreal, Canada
Why should we care about Multi-Resistant Bacteria? Clinical Impact & Public Health Implications	Prof. Stephan Harbarth<br>Infection Control Programme<br>University Hospitals of Geneva<br>University of Geneva, Switzerland
Antimicrobial Resistance in Foodborne Infections: Importance of the One Health approach	Dr. Simon Le Hello<br>Co-director National Reference Center E. coli/Shigella/Salmonella<br>Institut Pasteur, France
FoodNet Canada: Why is this important?	Prof. Andre Ravel<br>Faculty of Veterinary Medicine<br>University of Montreal, Canada

**Section 4 - Zoonotic Neglected Infectious Diseases**	
**Video-lecture**	**Expert and Affiliation**
Neglected Tropical Diseases & Zoonosis: Challenges & Solutions	Dr. Bernadette Abela-Ridder<br>Team Leader, Neglected Zoonotic Diseases<br>Department of the Control of Neglected Tropical Diseases<br>World Health Organization (WHO)
Towards Rabies Elimination: The case of Kuazulu-Natal (South Africa)	Kevin Le Roux<br>Rabies Project Manager<br>Department of Agriculture and Rural Development<br>KwaZulu-Natal, South Africa
Major Research Challenges & Opportunities in the fight against Rabies	Dr. Hervé Bourhy<br>Unit Lyssavirus dynamics and host adpatation,<br>WHO Collaborating Centre for Research on Rabies,<br>National Reference Centre for Rabies,<br>Institut Pasteur, France
Research & Innovation: Examples from Institut Pasteur’s fight against Rabies	Dr. Hervé Bourhy<br>Unit Lyssavirus dynamics and host adpatation,<br>WHO Collaborating Centre for Research on Rabies,<br>National Reference Centre for Rabies,<br>Institut Pasteur, France

Ecodog: A One health and Ecohealth approach to Rabies	Prof. Andre Ravel<br>Faculty of Veterinary Medicine<br>University of Montreal, Canada
One Health fight against Leishmaniasis	Prof. Afif Ben Salah<br>Head, Department of Medical Epidemiology<br>Institut Pasteur, Tunisia
Zoonotic Tuberculosis in the WHO End TB Strategy	Dr. Mario Raviglione<br>Director<br>Global TB Programme<br>World Health Organization (WHO)<br>Dr. Anna Dean<br>Global TB Programme<br>World Health Organization (WHO)

Section 5 - Conflicts & Injuries

Video-lecture	Expert and Affiliation
Protected Areas: How to manage Human-Wildlife-Domestic Animals Interface?	Dr. Geoffroy Mauvais<br>Program on African Protected Areas and Conservation (PAPACO)<br>International Union for Conservation of Nature (IUCN)
Snake-bites: A Neglected Global Health Challenge	Prof. François Chappuis<br>Head of the Division of Tropical and Humanitarian Medicine<br>University Hospitals of Geneva<br>University of Geneva, Switzerland
Snake-bites in Sub-Saharan Africa: A priority for "Doctors Without Borders"	Dr. Gabriel Alcoba<br>*Médecins sans Frontières* and<br>Division of Tropical and Humanitarian Medicine<br>University Hospitals of Geneva, Switzerland
Impact of Snake-bites in Livestock: The Case of Costa Rica	Dr. Maria Herrera<br>Instituto Clodomiro Picado<br>San Jose, Costa Rica

Section 6 - Innovation & Opportunities

Video-lecture	Expert and Affiliation
Citizen Science & Crowdsourcing: Opportunities & Challenges in Health	Prof. François Grey<br>Partnership Coordinator, Citizen Cyberlab<br>University of Geneva, Switzerland
Digital Epidemiology: Opportunities & Challenges in Global Health	Prof. Marcel Salathe<br>Head of the Laboratory of Digital Epidemiology<br>School of Life Sciences<br>Ecole Polytechnique Fédérale de Lausanne (EPFL), Switzerland

Section 7 - Health Benefits at the Human-Animal-Ecosystem Interface

Video-lecture	Expert and Affiliation
What is Environmental Health? A Historical overview	Dr. Jean Simos<br>Institute of Global Health<br>Faculty of Medicine<br>University of Geneva, Switzerland
Ecosystem Services & Health	Dr. Martin Schlaepfer<br>Institute of Environmental Sciences<br>University of Geneva, Switzerland
Growing with Nature: Implications for Child Health & Allergies	Dr. Caroline Roduit<br>Children's Hospital<br>Allergy Research Group<br>University of Zurich, Switzerland
Biodiversity as a Source of Food for Humans and Animals: Socio-cultural Determinants of Food Habits	Dr. Estelle Fourat<br>Research Centre on Work, Organizations and Policies<br>CERTOP<br>University of Toulouse – Jean Jaurès, France
Companion Animal Ownership: Benefits for Human and Animal Health	Dr. Dennis Turner<br>Director<br>Institute for Applied Ethology and Animal Psychology<br>Horgen/Zurich, Switzerland

Companion Animals & Economic Benefits of One Health	Dr. Dennis Turner<br>Director<br>Institute for Applied Ethology and Animal Psychology<br>Horgen/Zurich, Switzerland

Section 8 - Management of Ecosystems under Global Changes: Implication for Human Health	
**Video-lecture**	**Expert and Affiliation**
Increasing Health Sector Resilience to Climate Change and Disaster Risk: the Value of Biodiversity and Ecosystems	Dr. Tedros Adhanom Ghebreyesus<br>Director-General<br>World Health Organization (WHO)
Connecting Global Priorities: Biodiversity and Human Health	Ms. Cristina Romanelli<br>UN Convention on Biological Diversity (CBD) - World Health Organization (WHO) joint programme
Harnessing Biodiversity to Maximize Health Co-benefits in Agroecosystems	Dr. David Cooper<br>Deputy Executive Secretary<br>UN Convention on Biological Diversity (CBD)
Biodiversity and Human Health Linkages: Concepts, Determinants, Drivers of Change and Approaches to Integration	Dr. Diarmid Campbell-Lendrum<br>Department of Public Health and Environment<br>World Health Organization (WHO)
Global Overview of the United Nations Decade of Action on Nutrition	Dr. Francesco Branca<br>Department of Nutrition for Health and Development<br>World Health Organization (WHO)
Food Systems, Nutrition and Climate Change: The Need for a Cross-sectoral Approach	Ms. Lina Mahy<br>Department of Nutrition for Health and Development<br>World Health Organization (WHO)
Agrobiodiversity for Healthy, Diverse Diets and Food Systems. Case Studies from Brazil, Turkey, Kenya and Sri Lanka	Dr. Daniela Moura<br>National project lead<br>Biodiversity for Food and Nutrition, Brazil
Sustainable Oceans Management and Health	Dr. Jake Rice<br>Senior Scientist Emeritus<br>Department of Fisheries and Oceans, Canada
Sustainable Management of Agroecosystems and Health	Dr. Scott Newman<br>Senior Animal Health & Production Officer<br>Regional Office for Africa<br>Food and Agriculture Organization of the United Nations<br>Accra Ghana
The Role of Sustainably Managed Seascapes/Landscapes in Health and Nutritional Outcomes	Dr. Christopher Golden<br>Assistant Professor of Planetary Health and Nutrition<br>Harvard T.H. Chan School of Public Health, Boston
Gender and Health in the Face of Global Environmental Change	Ms. Elena Villalobos Prats<br>Department of Public Health and Environment<br>World Health Organization (WHO)
