# Supplementary material for: One Health education in Kakuma refugee camp (Kenya): From a MOOC to projects on real world challenges
Source: One Health. 2020 Aug 20;10:100158. doi: 10.1016/j.onehlt.2020.100158 (PMC7439830; doi:10.1016/j.onehlt.2020.100158)
Supplement: Appendix B — Survey of Kakuma Students and On-site Facilitators [file mmc2.docx]

**Appendix B. Survey of Kakuma students’ and on-site facilitators’ satisfaction**

Date: 27 January 2018

Online questionnaire

**Questions included in the satisfaction survey of Kakuma students at the end of the module 1 (Google Form):**

1. Please describe a previous experience that contributed to your motivation to apply for this course?

2. What aspect of the course do you find the most interesting and helps to maintain your motivation?

3. ln relation to the One health area, what do you hope to achieve in the future?

4. Please describe 2 positive and 2 negative aspects about pets, wildlife and livestock

5. Would you recommend the MOOC experience to your friends who didn't apply? If yes, how would you convince them to apply?

**Questions included in the survey of on-site facilitators at the end of the module 1
(Google Form):**

1. How would you qualify the general level of motivation of the students: did you expect more/less commitment? Were you impressed/disappointed or did you expect it?

2. Did you notice any improvements/actions made by the students in the community (related to knowledge they acquired in the MOOC)?

3. From your « facilitator perspective », did you notice something that didn’t work out (difficulties of a student for example)?
