# Supplementary material for: One Health education in Kakuma refugee camp (Kenya): From a MOOC to projects on real world challenges
Source: One Health. 2020 Aug 20;10:100158. doi: 10.1016/j.onehlt.2020.100158 (PMC7439830; doi:10.1016/j.onehlt.2020.100158)
Supplement: Appendix C — Motivations and Profiles of Applicants and Students [file mmc3.docx]

**Appendix C. Motivations and Profiles of Applicants and Students**

**a) Applicants’ motivations to take the One Health educational programme**

| **Applicants' motivations to take the One Health educational programme** | **Number of Applicants (Total=67)** | **%** |
| --- | --- | --- |
| Gain new knowledge and skills | 35 | 52 |
| Help own community and society at large | 21 | 31 |
| Global Health fits own career aspirations | 18 | 27 |
| Share knowledge/create awareness with community/other refugees | 11 | 16 |
| The importance of infectious diseases in the camp, own country or Africa | 10 | 15 |
| Prevent/fight diseases | 9 | 13 |
| The curriculum meets their qualification and professional experience | 6 | 9 |
| Desire for higher learning | 5 | 7 |
| Improve the health of the community and the world | 5 | 7 |
| Improve animal health and welfare | 4 | 6 |
| Opportunity to team up with scholars across the world and disciplines | 3 | 4 |
| Change own life/make future more brighter | 3 | 4 |
| Save human/animal life | 2 | 3 |
| Not specified | 2 | 3 |

**b) Profiles of Applicants and Students**

|  | **Applicants to Module 1 (N=67)** | |
| --- | --- | --- |
|  | n | % |
| **Gender** |  |  |
| Male | 45 | 67.2 |
| Female | 15 | 22.4 |
| Not specified | 7 | 10.4 |
| **Age group (years)** |  |  |
| 18-23 | 22 | 32.8 |
| 24-29 | 27 | 40.3 |
| 30-35 | 11 | 16.4 |
| 36-40 | 2 | 3.0 |
| > 40 | 1 | 1.5 |
| Unknown | 4 | 6.0 |
| **Age, mean+/-SD (range)**  25.9+/-4.9 (19-41) | | |

|  | **Students selected  for Module 1 (N=15)** | | **Students not selected  for Module 1 (N=52)** | |
| --- | --- | --- | --- | --- |
|  | n | % | n | % |
| **Gender** |  |  |  |  |
| Male | 9 | 60.0 | 36 | 69.2 |
| Female | 6 | 40.0 | 9 | 17.3 |
| Not specified | 0 | 0.0 | 7 | 13.5 |
| **Age group (years)** |  |  |  |  |
| 18-23 | 4 | 26.7 | 18 | 34.6 |
| 24-29 | 8 | 53.3 | 19 | 36.5 |
| 30-35 | 1 | 6.7 | 10 | 19.2 |
| 36-40 | 2 | 13.3 | 0 | 0.0 |
| > 40 | 0 | 0.0 | 1 | 1.9 |
| Not specified | 0 | 0.0 | 4 | 7.7 |
| **Age, mean+/-SD (range)**  26.6+/-5.5 (19-38) | | | 25.7+/- 4.7 (19-41) | |
| *P* 0.598 | | | | |

|  | **Students who passed Module 1 (N=6)^a^** | | **Students who did not pass Module 1 (N=8)^a^** | |
| --- | --- | --- | --- | --- |
|  | n | % | n | % |
| **Gender** |  |  |  |  |
| Male | 4 | 66.7 | 5 | 62.5 |
| Female | 2 | 33.3 | 3 | 37.5 |
| Not specified | 0 | 0.0 | 0 | 0.0 |
| **Age group (years)** |  |  |  |  |
| 18-23 | 3 | 50.0 | 1 | 12.5 |
| 24-29 | 3 | 50.0 | 4 | 50.0 |
| 30-35 | 0 | 0.0 | 1 | 12.5 |
| 36-40 | 0 | 0.0 | 2 | 25.0 |
| > 40 | 0 | 0.0 | 0 | 0.0 |
| **Age, mean+/-SD (range)**  23.6+/-3.5 (19-28) | | | 29.0+/- 6.2 (21-38) | |
| *P* 0.046 | | | | |

SD: Standard Deviation

^a^ number of students out of 14 (one female dropped out)

*P*: Method: Welch Two Sample t-test; Alternative :two.sided
