# Supplementary material for: One Health education in Kakuma refugee camp (Kenya): From a MOOC to projects on real world challenges
Source: One Health. 2020 Aug 20;10:100158. doi: 10.1016/j.onehlt.2020.100158 (PMC7439830; doi:10.1016/j.onehlt.2020.100158)
Supplement: Appendix D — Focus Group Discussion [file mmc4.docx]

**Appendix D. Focus Group Discussion**

Date: 21 February 2019

Location: Kakuma refugee camp – Kenya

Participants: Jade Mason (MGH student) and 5 Kakuma students

Main topics covered:

A. What was their experience overall while following this course?

B. What was the interaction with the students in Geneva like?

C. What was the students experience with the project based learning used in the course?

D. How do the students see their role moving forward?

A. When asked “what was their experience overall while following this course?” students spoke about how overall they enjoyed the content in the MOOC and the interaction with students in Geneva. As the session was framed as an opportunity to give constructive feedback to improve the course in the future students identified four main issues.

1. Access to content;
2. Understanding of content;
3. Flexibility; and
4. Additional content.
5. Access to content can be broken down into two main issues (i) issues around accessing content from the USB that contained the MOOC (ii) issues around internet access.

(i) Students highlighted that they needed to have access to a laptop or computer to have the course content and only two out of the four students had a laptop/tablet. Furthermore, consuming the course content off- online meant students were unable to access the transcripts of the MOOC videos, learners spoke about the challenges of understanding some experts, and that the transcripts of videos could help them to understand certain topics better as well as act as a study material.

(ii) Students spoke about the difficulties of internet connectivity in the camp, many learners found going online to complete the weekly quizzes was difficult. Furthermore, those students that did not have access to a laptop or tablet and therefore could not use the USB (when not in the InZone learning hub) could only access and download content when they had internet. When asked about using InZone’s hub students felt the internet at the hub was unreliable. During this discussion the cost of data was also brought up.

1. When speaking about the understanding of content all students wanted additional learning material such as text books and written guides, students felt this would help them with their understanding of the course content. In terms of the one on one teaching in Kakuma before their exam, students said that they would have liked to have revised all the course (6 out of the 8 weeks were covered during revision) and also go further in-depth in the revision of certain topics. The moderator asked if rather than one day of revision two days was allocated, all learners felt like this would be beneficial.
2. Flexibility issues related to accessing the InZone hub was another issue raised, particularly by those students that worked full time or had other commitments, they felt that the available times to access the hub were too restrictive. Students felt that many of the issues they face would be resolved if they all had tablets, to work remotely from home.
3. Learners were asked if there were certain topics that were not covered in the MOOC that they would have liked to be included in the future. Students spoke about the following

- Scorpions; and
- Climate change.

B. Learners in Kakuma were asked about their experience interacting with the learners in Geneva. While, again feedback was overall positive, one student wanted to have the MOOC forum and the WhatsApp tutoring group to be more aligned so they could both work in tandem as learning and tutoring tools.

C. Learners were also asked to share their experiences working and interacting with their groups in Geneva as part of the project based learning component of the course. Students had different experiences, two were very happy about their collaborations with the groups in Geneva, while two students felt that the group work was one sided, with students in Geneva asking the Kakuma student questions about the health issue assigned to them, but not involving the student in the project in more meaningful ways. Kakuma learners wanted to have more responsibility in the project e.g. writing certain parts of the proposal.

D. Finally, learners were asked about where they see their roles moving forwards, students saw themselves working on the community based epidemiological surveillance system for malaria as well as assisting the next cohort of students. Students also spoke about wanting to see more student making it through the two-year course.
